# Use of the Neurological Pupil Index to Predict Postoperative Visual Function After Resection of a Tuberculum Sellae Meningioma: A Case Report

**DOI:** 10.7759/cureus.5998

**Published:** 2019-10-25

**Authors:** Kunal P Raygor, Philip V Theodosopoulos

**Affiliations:** 1 Neurological Surgery, University of California, San Francisco, USA

**Keywords:** neurological pupil index, tuberculum sella meningioma, optic apparatus, oculomotor reflex

## Abstract

The Neurological Pupil index (NPi) is a standardized method for evaluating pupil reactivity that removes inter-examiner variability. Changes in the NPi can predict clinical deterioration in patients with traumatic brain injury (TBI); however, its use to predict visual impairment after the resection of parasellar meningiomas has not been described. A 71-year-old female underwent a modified expanded bifrontal craniotomy for resection of a 3.1 cm tuberculum sella meningioma that caused compression of the optic chiasm and resulted in left temporal and right superior temporal visual field deficits. Postoperatively, she lost vision in the right eye. Pupillometer measurements demonstrated an asymmetrically low NPi at that time, which improved to normal prior to partial vision recovery. The average NPi in the right pupil was 1.67 during the time of vision loss compared to 3.47 in the left pupil (p=1.7x10^-10^). Statistical analysis was performed with the Student’s t-test and the significance level was set at p-value < 0.01. Resection of parasellar meningiomas is challenging because of the proximity of the optic apparatus. We report a case of unilateral vision loss after resection of a tuberculum sella meningioma in which the impaired eye’s NPi value correlated closely with visual function. NPi values that decrease below 3 predict spikes in intracranial pressure in TBI patients; similarly, increases in the NPi value above 2.5-3 predict improvement in vision in the case reported here. By monitoring the proximal portion of the oculomotor reflex, the NPi can be a marker of visual impairment after surgery.

## Introduction

The Neurological Pupil index™(NPi®) standardizes the evaluation of pupil reactivity. Using the NPi-200 Pupillometer System (NeurOptics®, Irvine, California), the NPi (measured on a 0-5 scale) is derived by a proprietary algorithm; values closer to 0 represent “sluggish” or “abnormal” pupils and values closer to 5 represent normally reactive pupils. The NPi is used in trauma settings to quantify pupil reactivity, and changes in NPi over time predict clinical deterioration [1-3]. Pupil reactivity may also predict vision changes in patients undergoing craniotomy for the resection of tumors adjacent to the optic nerves, including parasellar meningiomas. The case presented here shows that improving NPi after postoperative vision loss precedes clinical visual improvement in a patient who underwent resection of a tuberculum sella meningioma.

## Case presentation

**Figure 1:**
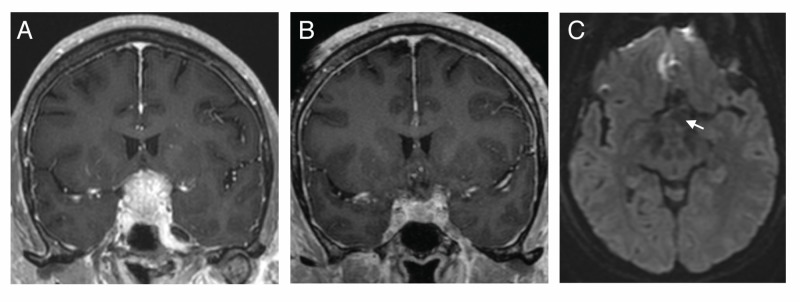
Characteristic pre- and postoperative magnetic resonance imaging (MRI) A) Preoperative coronal T1 post-gadolinium MRI scan revealing homogeneously enhancing mass encasing bilateral internal carotid arteries and causing optic nerve/chiasm elevation. B) Postoperative coronal T1 post-gadolinium MRI scan revealing near-total resection. C) Postoperative axial diffusion-weighted sequence revealing no definitive reduced diffusion along the optic nerves or chiasm (denoted by the white arrow).

A 71-year-old female presented with 2.5 years of visual loss in her left eye and was found on Humphrey visual field testing to have left temporal and right superior temporal visual field deficits. An MRI scan revealed a 3.1 cm tuberculum sella meningioma encasing bilateral internal carotid arteries and causing elevation of the optic chiasm (Figure 1A). She underwent modified extended bifrontal craniotomy for tumor resection [4]. Postoperative MRI revealed expected postsurgical findings without a compressive hematoma or optic nerve/chiasm infarction (Figures 1B-1C).

Postoperative course

**Figure 2:**
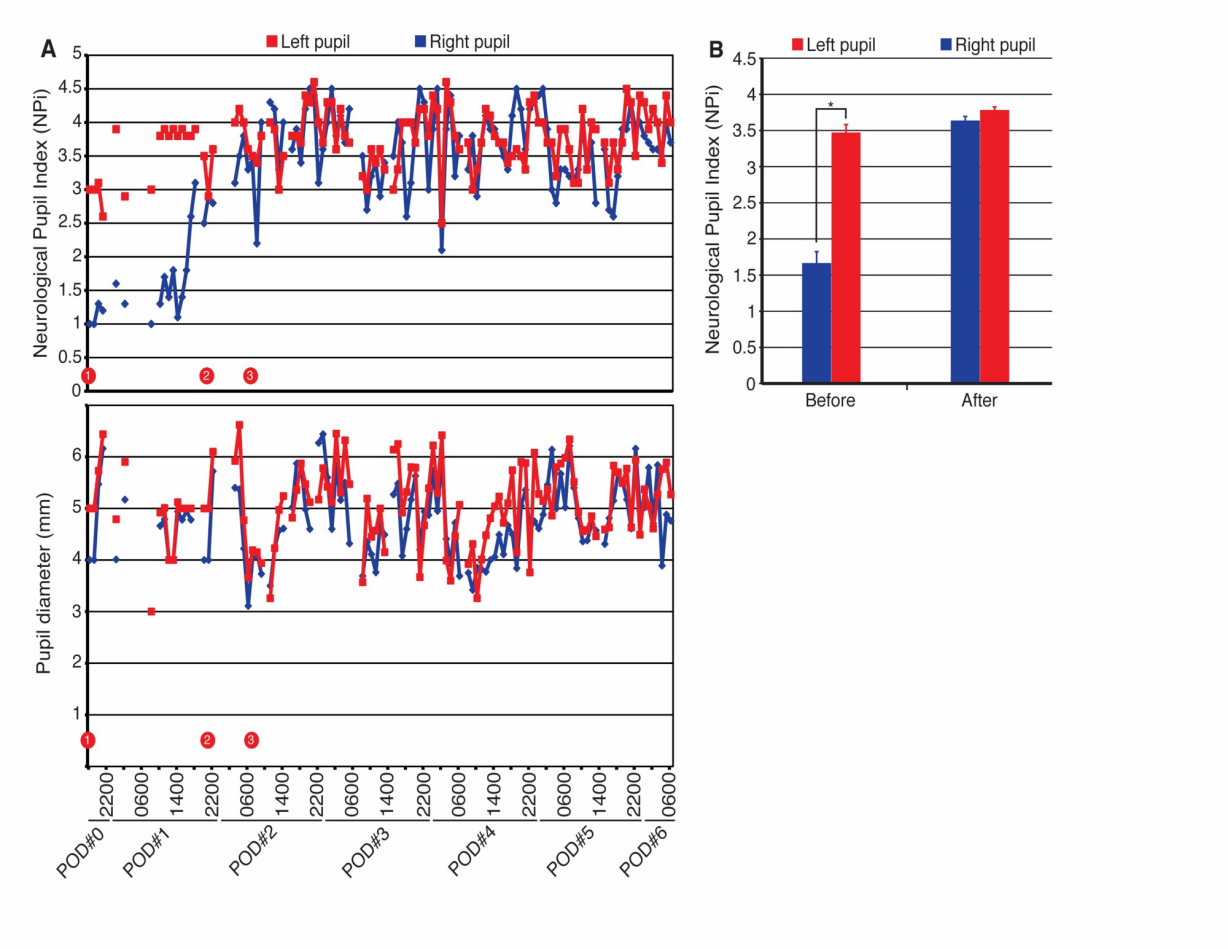
Neurological Pupil index (NPi) can be used as a marker of visual impairment A) Plot of NPi values (top panel) and pupil diameters (bottom panel) for right (blue) and left (red) pupil after a visual deficit was seen in the postoperative setting. Blank areas represent gaps in the electronic medical record. Time point 1 refers to the moment at which the patient lost vision in her right eye. Time point 2 refers to the moment at which the patient first regained the ability to see fingers and objects in her right eye. Time point 3 refers to the moment at which she was able to read a name badge at a distance of approximately 2 feet. B) Average NPi value for right and left pupils before time point 2 and after time point 2. *represents a highly statistically significant difference (p=1.7 x10-10).

**Figure 3:**
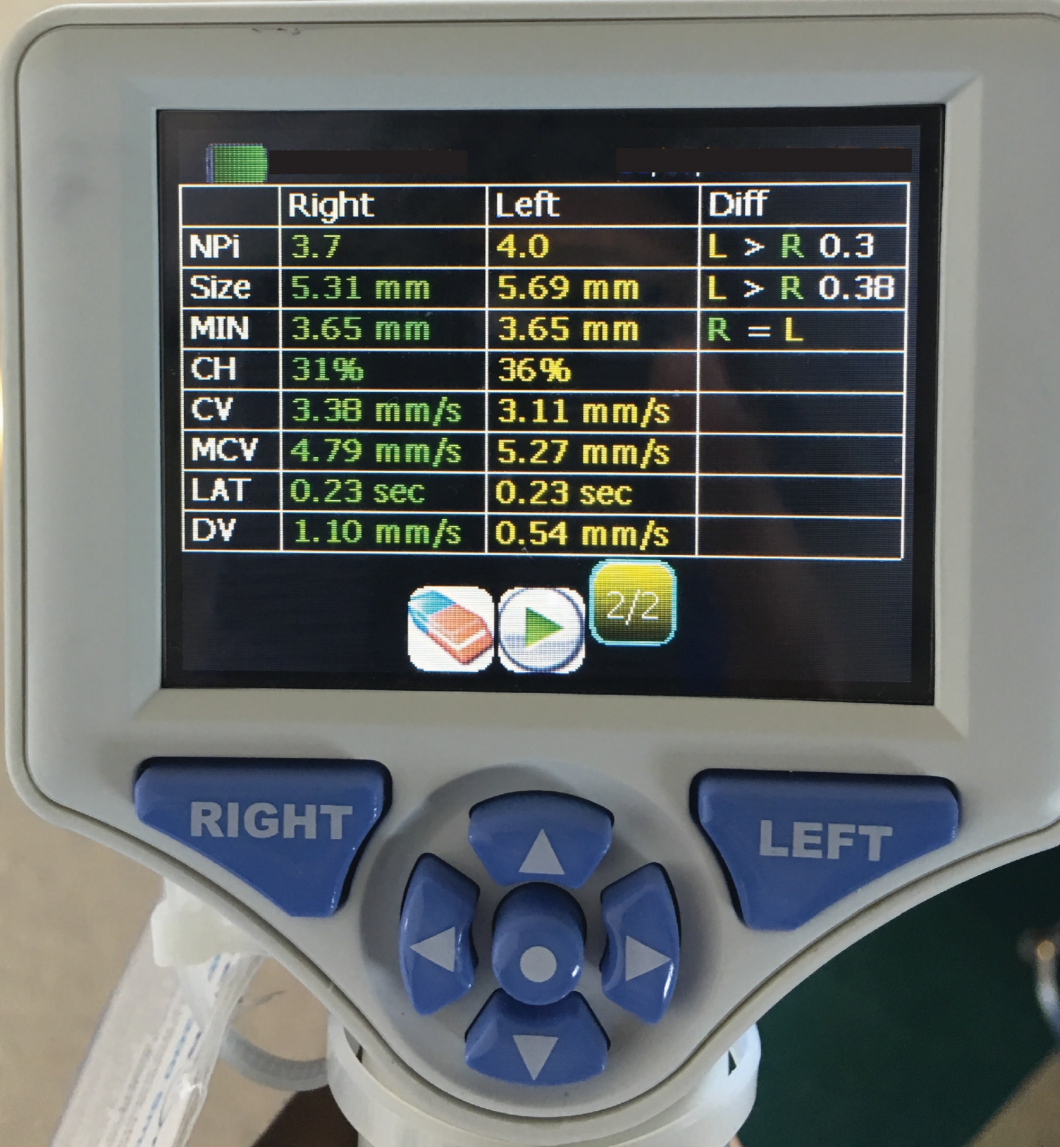
NPi-200 Pupillometer console Photograph of NeurOptics® NPi-200 Pupillometer console demonstrating values of pupil measurements performed after time point 2. NeurOptics® NPi-200 Pupillometer: NeurOptics®, Irvine, California

Immediately after surgery, the patient’s vision was at her preoperative baseline and pupils were briskly reactive on the standard penlight exam. Within a few hours of recovering in the intensive care unit (ICU), however, the patient lost vision in her right eye, retaining minimal light perception. At that time, the right pupil was sluggishly reactive with an afferent pupillary defect; thereafter, pupil exams were performed using a pupillometer. The NPi values of the right and left pupils were 1 and 3, respectively; the pupil diameter was 4 and 5 mm, respectively (time point 1, Figure 2A). To treat presumed ischemia to the optic apparatus, the patient’s systolic blood pressure (SBP) was elevated with vasopressors and intravenous normal saline. Bilateral NPi and diameter values were recorded hourly and are plotted in Figure 2A (top and bottom panels, respectively). The NPi in the right pupil exceeded 2.5 a few hours before the patient began seeing fingers and objects in her right eye again (Figure 2A, time point 2). Time point 3 in Figure 2A refers to the moment at which she was able to read a name badge at a distance of ~2 feet. The average NPi in the right pupil was 1.67 as compared to 3.47 in the left pupil between time points 1 and 2 (p=1.7x10-10) (Figure 2B). On the other hand, NPi values after time point 2 were more similar (3.64 vs 3.78, p= 0.04). Figure 3 demonstrates a representative image of the pupillometer console after time point 2 with all pupil variables. Once the patient’s vision stabilized, blood pressure goals were liberalized. She was ultimately discharged home in good condition and at her three-month follow-up appointment, was noted to have clear vision in her right eye with her baseline visual field deficits.

## Discussion

The close proximity of the visual apparatus makes resection of tuberculum meningiomas challenging; visual outcomes are crucial to determining the safety and efficacy of tumor resection. Many microsurgical series have described visual function improvement rates of 40%-90%, though they also report visual deterioration in up to 30% of patients [5-12]. Avoiding any interruption of the vascular supply to the optic nerves and chiasm (typically via the superior hypophyseal arteries) is of paramount importance; the expanded bifrontal approach is particularly equipped to preserve this vascular supply [4,12]. Any manipulation of the nerves or vasculature, however, can lead to postoperative visual impairment. The case presented here shows that the NPi can be a marker of visual impairment and subsequent recovery in cases of presumed reversible vascular ischemic causes of postoperative visual loss.

Monitoring the pupillary light reflex inherently monitors optic nerve function. The afferent limb of this reflex arc involves the transmission of information from photosensitive retinal ganglion cells to pretectal nuclei via the optic nerves. Unilateral optic nerve injury classically leads to an afferent pupillary defect (APD), which was seen in this case. Unfortunately, the pupillometer cannot detect APDs because it only measures pupil reactivity in the eye in which light stimulation is provided. Even so, manual examination of pupil reactivity has high inter-examiner variability compared to pupillometry measurements [3,13]. This variability is compounded by the use of sedating medications, which makes it harder to see changes in already constricted pupils. The use of the pupillometer can thus standardize this process. The NPi takes into account the following variables, which are displayed on the pupillometer console in Figure 3: Size (resting pupil diameter), MIN (minimal pupil diameter during constriction), CH (percent change in diameter with constriction), CV (average constriction velocity), MCV (maximum constriction velocity), LAT (latency between initiation of light stimulation and onset of pupillary constriction), and DV (average dilation velocity). Those variables are then compared to reference values taken from subjects with intact vision. NPi values ≥3 are considered “normal,” though NPi values closer to 5 are considered “more briskly reactive” than those closer to 3. Our report demonstrates that NPi values are statistically significantly lower in impaired versus unimpaired eyes postoperatively and that the values return to normal with vision improvement (Figures 2A-2B). The pupil diameter was largely similar between time points 1 and 2 (Figure 2A), but the other pupil variables were not trended longitudinally.

Prior studies demonstrate that NPi changes can predict intracranial pressure (ICP) changes in TBI patients. In particular, NPi values <3 correlate with ICP spikes >20 mmHg that can affect the oculomotor nerve during uncal herniation (thereby affecting the efferent limb of the pupillary light reflex) [1-2]. In the case presented here, the NPi of the right pupil crossed a threshold between 2.5 and 3 prior to the patient being able to see fingers in her right eye (time point 2, Figure 2A). Thus, the utility of measuring NPi values is in following their trends over time. Our report suggests that NPi values that increase beyond a threshold of 2.5-3 may predict visual improvements in patients undergoing parasellar tumor resections. The NPi, in this case, may function as a marker of optic nerve function as opposed to being predominantly a marker of oculomotor nerve or Edinger-Westphal nucleus function in the case of patients with TBI.

Future prospective studies should determine the overall effectiveness of pupillometry measurements in predicting vision improvement in patients undergoing tuberculum sellae meningioma resections. Though NPi measurements are limited by the fact that they only measure a single pupil at a time (and thus cannot quantify APDs), our report suggests that trending NPi values can predict visual outcome in cases of suspected unilateral optic nerve ischemia/injury after surgical resection of parasellar tumors.

## Conclusions

Much like its predictive abilities in TBI patients, NPi values may predict vision improvement after suspected vascular-ischemic causes of vision loss following resection of tuberculum sellae meningiomas. Unlike in cases of TBI, we propose that NPi abnormalities likely reflect the afferent arc of the pupillary light reflex in the case described here. Future work should include cohort studies that more thoroughly examine the predictive capacity of pupillary measurements.
